# The Effects of Pectin Structure on Emulsifying, Rheological, and *In Vitro* Digestion Properties of Emulsion

**DOI:** 10.3390/foods11213444

**Published:** 2022-10-30

**Authors:** Xixiang Shuai, Jun Chen, Qi Liu, Haolan Dong, Taotao Dai, Zhaoying Li, Chengmei Liu, Risi Wang

**Affiliations:** 1 State Key Laboratory of Food Science and Technology, Nanchang University, Nanchang 330047, China; 2 School of Food Science and Engineering, Jiangxi Agricultural University, Nanchang 330045, China

**Keywords:** pectin, emulsifying properties, rheology, in vitro digestion, degree of methoxylation

## Abstract

Pectin, a complex hydrocolloid, attracts extensive attention and application stemming from its good emulsification. However, the source of emulsification remains a conundrum. In this experiment, the structures of six kinds of commercial pectin, including LM 101 AS (***101***), LM 104 AS (***104***), 121 SLOW SET (***121***), YM 150 H (***150***), LM 13 CG (***13CG***), and β-PECTIN (***β-P***) were determined, and the effects of pectin structure on emulsion emulsification, rheology and in vitro digestibility were studied. The results showed that the ***β-P*** pectin contained a higher content of protein, ferulic acid, and acetyl and had a lower interfacial tension; this pectin-stabilized emulsion exhibited a smaller droplet size and superior centrifugal and storage stability. The results showed that ***β-P*** pectin had higher contents of protein, ferulic acid, and acetyl and lower interfacial tension than other pectins, and its stabilized emulsion exhibited smaller droplet size and superior centrifugation and storage stability. Furthermore, the emulsion formed by the pectin with high molecular weight and degree of methoxylation (DM) had a higher viscosity, which can inhibit the aggregation of emulsion droplets to some extent. However, the DM of pectin affected the charge and digestion behavior of pectin emulsion to a great extent. The smaller the DM, the more negative charge the emulsion carried, and the higher the release rate of free fatty acids. The results provided a basis for the rational selection and structural design of the pectin emulsifier.

## 1. Introduction

Pectin, a kind of complex linear polymer polysaccharide, is widely found in plant cell walls and is mainly composed of three structures, namely homogalacturonan, rhamnogalacturonan I, and rhamnogalacturonan II [1,2]. Pectin is commonly used in the food industry as a gelling agent, stabilizer, and thickener. In 2020, the global production of pectin was approximately 70,000 tons, with sales exceeding USD 1.25 billion, making it one of the most promising natural hydrocolloids. Recently, pectin has been increasingly accepted as an emulsifier for emulsion stabilization due to its potential emulsification, biodegradability, and biocompatibility [3,4]. Emulsions are thermodynamically unstable exhibiting coalescence, flocculation, Ostwald ripening, creaming, and other phenomena [5]. During emulsion preparation, pectin can rapidly adsorb to the interface resulting in short-term stability, and the carbon chain of pectin can be extended around the droplets to slow collision and maintain long-term stability. Recently, many pectins derived from natural plants have been reported to have good emulsifying properties, such as apple, citrus, sugar beet, potato pulp [6], *Nicandra physaloides* (Linn.) *Gaertn* seeds [7], and Pomegranate peel [8]. However, it is difficult to pinpoint the source of emulsifying properties of pectin, arousing scientific discussion invariably.

As a hydrophilic colloid, the formation of stable emulsions of pectin is attributed to the hydrophobicity of proteins, acetyl groups, methyl groups, and ferulic esters in pectin at the oil-water interface [4,9]. In addition, the protein content, molecular weight (M_w_), degree of esterification (DE), and monosaccharide side chain proportion of pectin have been reported to affect emulsifying properties. Although these structural factors have been investigated, the relationship between pectin structure and emulsifying properties remains controversial. It was suggested that the interfacial activity of pectin was related to the presence of protein, which acted as a hydrophobic anchor to promote the adsorption of the pectin chain at the interface, thus reducing the interfacial tension [10]. Whereas more studies were inclined to believe that protein is not the controlling factor of the stability of pectin emulsion, and high protein concentration or presence of protein cannot ensure good emulsification performance [11,12,13]. It was indicated that the acetyl group could significantly improve the emulsifying performance of pectin, especially in the case of low protein content [14,15], but optimistic opinion concluded that citrus pectin, which is low in acetyl, may have an interesting emulsifying capacity [16]. In addition, a quadratic equation (y = −0.0037(x − 52.61)^2^ + 11.97 or y = −0.0076(x − 53.95)^2^ + 7.12) between the particle size of milk droplets and the degree of esterification have been found by investigating sugar beet pectin as well as apple and citrus pectin with different degree of methoxylation [17]. Pectin emulsions with moderate esterification degrees had the largest droplet size, but other investigations found block-wise de-esterified pectin had lower interfacial tension [18]. The esterification degree of pectin was reduced by methyl esterase from 67% to 7%, altering the emulsion droplet size and interfacial tension slightly [14]. As reported, pectin with higher molecular weight was more conducive to improving the stereoscopic stability of milk droplets [19], while Schmidt et al. [20] believed that molecular weight did not directly affect the emulsification. However, as far as we know, the effects of pectin structure on emulsion emulsification, rheology, and in vitro digestibility have not been systematically discussed.

Hence, this paper aims to (1) characterize the structure of six kinds of pectin and prepare the emulsion, (2) investigate the effects of pectin structure on emulsification, rheology, and in vitro digestibility of emulsion. This study will promote the application of pectin emulsifiers and especially provide a basis for the rational selection of emulsifiers in structure.

## 2. Materials and Methods

### 2.1. Materials

Six types of pectin samples with different structures, including LM 101 AS (101), LM 104 AS (***104***), 121 SLOW SET (***121***), YM 150 H (***150***), LM 13 CG (***13CG***), and β-PECTIN (***β-P***) were kindly provided by CP Kelco Company (Shanghai, China). Galacturonic acid monohydrate (≥97.0%), pepsin from porcine gastric mucosa (400 units/mg), dextran (≥80.0%), porcine lipase (100–400 units/mg) and bile salt (cholic acid ≥60%) were purchased from Sigma-Aldrich Chemical Inc. (Shanghai, China). Soybean oil was obtained from a local supermarket (Nanchang, Jiangxi Province, China). All other reagents, including hydrochloric acid (36%), concentrated sulfuric acid (98%), ethanol (100%), sodium hydroxide (96.0%), ferric chloride (≥98.5%), and hydroxylamine hydrochloride (≥98.0%) were of analytical reagent grade. Self-made distilled water (Electrical conductivity: 3.8 us/cm, pH 6.98) was used in all experiments.

### 2.2. Structural Characterization of Pectin Samples

The galacturonic acid (GA) content, degree of methoxylation (DM), degree of amidation (DAm) of pectin samples, molecular weight (M_w_) of ***101***, ***104***, ***121***, ***150***, ***13CG***, and ***β-P*** had been characterized by *m*-hydroxybiphenyl method, titration method and high-performance size exclusion chromatography (Agilent 1200, Agilent Technologies, Santa Clara, CA, USA) equipped with a refractive index detector, respectively. The experimental detail was described in previous research [21].

#### 2.2.1. Determination of Degree of Acetyl

The degree of acetyl (DAc) of pectin was characterized using the method of hydroxylamine colorimetry [22]. Briefly, 1 mL of 10 mg/mL pectin solution mixed with 5 mL 0.1 mol/L refreshed hydroxylamine hydrochloride and 5 mL of 1.5 mol/L NaOH solution. This reaction was performed at room temperature for 15.0 min. Then, 3.5 mL of 2 mol/L HCl solution was added to neutralize the excessive NaOH. After standing for 15 min, 10 mL of 0.37 mol/L ferric chloride (FeCl_3_) solution was added, and the reaction solution was supplemented with distilled water to 50 mL. Finally, the absorbance of the reaction solution was measured at the wavelength of 500 nm after 10 min of reaction. The same amount of distilled water was used to replace the pectin solution as the blank control. The *β*-D-5-acetyl glucose standard was used to draw the standard curve. Then, the DAc was calculated through the following equation (Equation (1)) [23].

(1) $$\begin{array}{c} \mathrm{DAc}\left(\%\right)=\frac{\mathrm{acetyl}\;\mathrm{content}\left(\%\right)\;÷\;43}{\mathrm{GalA}\left(\%\right)\;÷\;\left[\frac{\left(\%\mathrm{ME}\;\times \;190\right)+\left(\%\mathrm{NE}\;\times \;176\right)}{100}\right]}\times 100 \end{array}$$

where ME represents methyl-esterified anhydro galacturonic acid (M = 190 g/mol), NE refers non-esterified anhydro galacturonic acid (M = 176 g/mol), and acetyl content represent the percentage (*w/w*) of acetyl group (M = 43 g/mol) in the sample.

#### 2.2.2. Determination of Protein Content

The protein contents of pectin (***β-P***,***121***, ***150***, ***101***, ***13CG,*** and ***104***) were analyzed by automatic Kjeldahl apparatus. The detailed operation method has been reported by Liu et al. [7]. Protein content was calculated according to the following formula (Equation (2)).

(2) $$\begin{array}{c} X\;\left(\%\right)=\frac{\left(V_{1}-V_{2}\right)\;\times \;c\;\times \;0.014}{{m\;\times V}_{3}/100}\;\times \;F\;\times \;100 \end{array}$$

where X represents the protein content, and V_1_ and V_2_ denote the volume of HCl solution consumed in the sample and control, respectively. c refers to the concentration of HCl solution, m represents the weight of the sample, V_3_ denotes the volume of digestive liquid, and F refers to the conversion coefficient.

### 2.3. Dynamic Interface Characteristics

According to the method of Tamm et al. [24], the dynamic interface properties of different pectin at the water–oil interface were measured by the optical contact angle measuring instrument (OCA-20, Data-physics Instruments GmbH, Filderstadt, Germany) with the oscillator generator attachment. All experiments were conducted at ambient temperature (25 °C).

#### 2.3.1. Dynamic Interfacial Tension

The dynamic interfacial tension of pectin samples at the water–oil interface was analyzed by the method of drop shape analysis, according to Jia et al. [25]. The stainless-steel needle (outside diameter 1.65 mm, inside diameter 1.19 mm) connected to the electric injection control unit was inserted into the rectangular glass tank containing purified corn oil (0.9219 g/cm^3^), and then the pectin solution was injected using a syringe by the control unit. After 13 μL droplets were formed at the tip of the needle, the stainless-steel needle was allowed to be adsorbed at the water–oil interface for 180 min. The images of the droplet were continuously recorded and digitized through the Charged Coupled Device (CCD) video camera system (Filderstadt, Germany). The interfacial tension (γ) was calculated by the Young-Laplace equation using Equations (3) and (4):

(3) $$\begin{array}{c} \gamma =\frac{\Delta \rho \cdot g}{C} \end{array}$$

(4) $$\begin{array}{c} \frac{1}{x}\cdot \frac{d}{\mathrm{dx}}\left(x\cdot \sin\theta \right)=\frac{2}{b}-C\cdot Z \end{array}$$

where Δρ represents the density difference between two phases; g represents the acceleration of gravity; C represents a capillary constant; b represents the curvature radius of the droplet fixed point *P*; x and Z represent the vertical and horizontal coordinates of any point on the plane plan of the external contour of the droplet, respectively; and θ represents the angle of osculation of any point.

#### 2.3.2. Interfacial Dilatational Viscoelasticity

The interfacial dilatational viscoelasticity of pectin samples at the oil–water interface were analyzed by the method of drop shape analysis [25]. In brief, a drop of oil (13 μL) was formed at the tip of the needle, and then the tip was submerged into a cuvette with a 30 mL pectin sample (1.0%, *w/v*). The relationship between interfacial dilatational modulus (E) and time (120 min) was analyzed at 0.1 Hz and 10% deformation amplitude (ΔA/A_0_ = 0.1). The E was calculated according to Equations (5)–(7)

(5) $$\begin{array}{c} \sigma =\sigma 0\;\sin\;(\omega t+\delta ) \end{array}$$

(6) $$\begin{array}{c} A=A0\;\sin\;(\omega t) \end{array}$$

(7) $$\begin{array}{c} E=\frac{d\sigma }{d\;A/A}=\frac{d\pi }{d\;\ln\;A} \end{array}$$

where σ and σ0 represent the dilatational stresses at t and 0 min, respectively; δ denotes the phase angle between the stress and strain; and A and A_0_ refer to the drop’s surface area at t and 0 min, respectively.

### 2.4. Preparation of Emulsion Based on Pectin

The emulsion preparation was based on the method of the previous research [26] with some modifications. First of all, a coarse emulsion was prepared by blending 10% (*w/w*) soybean oil and 90% (*w/w*) pectin aqueous (1%, *w/w*) using a high-shear mixing device (Ultra Turrax, IKA T18 basic, Staufen, Germany) for 2 min at 12,000 rpm. Then, the coarse emulsion was further homogenized by a high-pressure microfluidizer (M-110EH30, Microfluidic Corp, Newton, MA, USA) 3 times at a pressure of 80 MPa. Finally, sodium azide (0.01%, wt%) was added to inhibit microbial growth of the emulsion. After preparation, the emulsions were kept at ambient temperature (25 °C) in the dark for 7 days, and any changes in their properties (appearance, mean diameter, particle size distribution, and zeta potential) were measured.

#### 2.4.1. Particle Characteristics

The particle size of emulsion samples was determined by laser light scattering (Mastersizer 3000, Malvern Instruments, Worcestershire, UK). The results were presented as the particle size distribution (PSD), volume-weighted mean diameter (d_4,3_), and surface-weighted mean diameter (d_3,2_). The appearance of all emulsions was pictured with a digital camera (D3500, Nikon, Nanjing, China).

#### 2.4.2. Measurement of Zeta Potential

The zeta potential of emulsion samples was measured by Zetasizer Nano-ZS90 (Malvern Instruments, Worcestershire, UK) according to the method of Li et al. [27]. Before analysis, all emulsions were diluted with buffer solutions of the same pH and ionic strength to avoid multiple scattering effects.

#### 2.4.3. Centrifugal Stability

The centrifugal stability of the six pectin emulsions was measured using LUMiSizer (L.U.M. GmbH, Berlin, Germany). The following parameters were used for measurement: rotational speed, 1320 g; performed time 7620 s; time interval, 30 s; temperature, 25 °C. The instability index was calculated by the SepView 6.0 software (LUM, Berlin, Germany), which is a dimensionless number between 0 and 1. “0” indicates no changes in particle concentration (very stable), and “1” indicates that the dispersion has completely phase separated (very unstable) [28].

#### 2.4.4. Rheological Properties

The rheological properties of the emulsion were determined using a rheometer (MCR 302, Anton Paar, Graz, Austria) equipped with a 50 mm parallel plate (PP50) and set the gap to 1.0 mm, according to the previous study [29,30]. The steady flow behavior of emulsion (apparent viscosity and shear stress) was measured at 25 °C as the shear rate increased from 0.01 to 100 s^−1^.

### 2.5. The Measurement of Free Fatty Acids under the In Vitro Digestion

The digestion properties of the pectin-based emulsion samples were determined according to the method reported previously with some modifications [31,32]. Briefly, all solutions were preheated to 37 °C prior to use, and in vitro digestion experiments were simulated at this temperature. Mouth phase: 10 mL of emulsion sample was mixed with 10 mL of simulated saliva fluid (SSF) containing 0.003 g/mL mucin, and the pH of the mixture was adjusted to 6.8 ± 0.01 with 0.1 mol/L NaOH solution, followed by incubation in a 37 °C water bath for 10 min. Stomach phase: 20 mL of simulated gastric fluids (SGF) containing 0.0032 g/mL pepsin was added to the above reaction solution, the pH was adjusted to 1.2 ± 0.01 with 2 mol/L HCl solution, and the mixture was then incubated at 37 °C for 2 h. Small intestine phase: after the stomach simulation reaction, the pH of the mixture was adjusted to pH 7.0 ± 0.01 with 2 mol/L NaOH solution, and then 1.5 mL salt solution (0.25 mol/L CaCl_2_ and 3.75 mol/L NaCl) and 3.5 mL bile salt solution (0.0536 g/mL) were added. Meanwhile, 200 μL lipase solution (0.5 g/mL) was added to start the intestine digestion reaction. Finally, an automatic titration unit (902 Titrando, Metrohm Inc., Herisau, Switzerland) was used to monitor and maintain the pH at 7.0 ± 0.01 for 2 h throughout the titration by adding 0.25 mol/L NaOH solution. The amount of free fatty acids (FFAs) released was calculated from the amount of NaOH needed for titration.

### 2.6. Statistical Analysis

All tests were performed in triplicate, and data were presented as means ± standard deviations. Statistical analysis was performed on SPSS 25.0 (SPSS Inc., Chicago, IL, USA) and Origin 2020 (OriginLab, Northampton, MA, USA). A one-way analysis of variance was used to analyze the significant difference (*p* < 0.05).

## 3. Results and Discussion

### 3.1. Structural Characteristics of Pectin

The structural characteristics of six types of pectin are shown in Table 1. The order of GA content in pectin ranged from high to low: ***13CG*** (87.73%) > ***121*** (82.29%) > ***β-P*** (81.02%) > ***150*** (78.33%) > ***101*** (70.17%) > ***104*** (65.59%). All the GA content of pectin was higher than 65% galacturonic acid unit, indicating that the quality of pectin met the standard of commercial according to the Food and Agriculture Organization (FAO) and European Union (EU). The M_W_ was reported to be an important structural characteristic of pectin to determine the conformation of pectin in solution, which can affect the forming of a protective layer on the surface of oil droplets and the spatial stability of emulsion [4]. The M_W_ range of pectin was from 527 kDa to 1273 kDa; 13cg pectin had the lowest M_W_ (527 kDa), while ***150*** pectin possessed of highest M_W_ (1273 kDa). The DE was a critical parameter that affected the emulsifying performance of the emulsion [17]. The order of DM content in pectin ranged from high to low: ***150*** (72.65%) > ***β-P*** (62.46%) > ***121*** (58.48%) > ***13CG*** (41.46%) > ***101*** (37.13%) > ***104*** (28.28%). Among these pectin samples, ***150***, ***β-P***, and ***121*** with more than 50% DE belong to the high methoxyl pectin (HMP) samples [2,30]. Pectin with different DAc affected the functionality, including emulsifying gel properties [33]. The DAc of pectin were ***β-P*** (3.03%), ***121*** (2.73%), ***150*** (2.61%), ***104*** (2.40%), ***101*** (2.33%), and ***13CG*** (2.31%), respectively. The results showed that the acetylation of pectin (2.31% to 3.03%) was not significantly different. The amidation of pectin is also an important characteristic that effected the rheological properties of pectin [34]. In our study, the acetyl content of pectin samples was ranked from high to low: ***104*** (20.63%) > ***101*** (14.89%). The other pectin samples, including ***121***, ***150***, ***13CG***, and ***β-P,*** were not amidated. Proteins are used as emulsifiers to promote the formation, improve stability and provide specific physicochemical properties to oil-in-water emulsions [35]. The protein content of pectin samples were 7.74% (***121***), 4.67% (***β-P***), 4.42% (***150***), 3.47% (***13CG***), 2.65% (***101***), and 1.90% (104).

### 3.2. Characteristics of Pectin at Water–Oil Interface

#### 3.2.1. Dynamic Interfacial Tension

**Figure 1:**
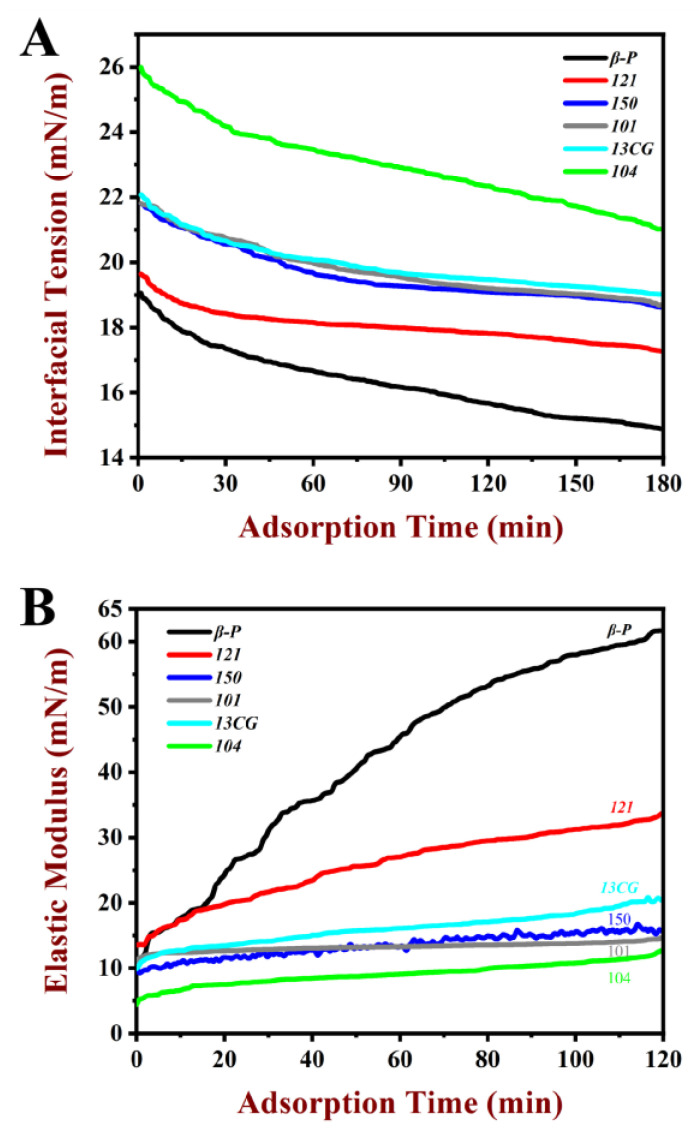
Characteristics of pectin at water–oil interface as a function of time of the pectin samples at 1% (*w/w*) concentration. (**A**) The dynamic interfacial tension of ***β-P***, ***121***, ***150***, ***101***, ***13CG,*** and ***104*** in the water–oil interfacial during the 180 min adsorption process. (**B**) The elastic modulus value of ***β-P***, ***121***, ***150***, ***101***, ***13CG,*** and ***104*** in the water–oil interfacial during the 120 min adsorption process. **Note: *β-P***, ***121***, ***150***, ***101***, ***13CG,*** and ***104*** were abbreviations of β-PECTIN (***β-P***), 121 SLOW SET (***121***), YM 150 H (***150***), LM 101 AS (***101***), LM 13 CG (***13CG***), and LM 104 AS (***104***), respectively, which were six types of pectin samples.

The interfacial tension (γ) of pectin at water–oil interface changes with time and is presented in Figure 1A. The γ value of all pectin samples decreased gradually with the increase in time, because the pectin was adsorbed on the interface as a surface-active substance. It was worth noting that the γ did not reach equilibrium within three hours. The γ decreased significantly in all systems from 0 to 20 min, which indicated the pectin was rapidly adsorbed on water–oil interface. From 20 to 180 min, the attenuation rate of γ decreases, which may be due to the stable adsorption of pectin on water–oil interface, but it was difficult to achieve a fully balanced state [36]. Therefore, the γ value at 180 min was used to present the γ. In Table 2, among the six pectin samples, the ***β-P*** had the lowest γ (14.9 mN/m), followed by ***121*** (17.27 mN/m). The ***104*** had the highest γ value (21.02 mN/m). As shown in Figure 1A, the γ value of ***β-P*** was lower than those of ***121***, ***150*** (18.62 mN/m), ***101*** (18.69 mN/m), ***13CG*** (19.02 mN/m), and ***104***, suggesting the ability to reduce γ was better than other pectin samples. The strongest ability of ***β-P*** pectin to reduce γ may be because of its high DE and protein content, as well as significantly high DAc and ferulic acid content [37]. Chen et al. [14] reported that the effect of functional groups on surface activity was in the following order: ferulic acid > ferulic acid–arabinogalactan–protein complexes > protein, which explained that although ***121*** pectin had the highest protein content, its surface tension was still higher than that of ***β-P***.

#### 3.2.2. Dilatational Rheological Properties

The elastic modulus value of pectin at the water–oil interface during the 120 min adsorption process is shown in Figure 1B. The elastic modulus value of pectin samples increased gradually during the adsorption process because the pectin was adsorbed on the interface as a surface-active substance, which was similar to the results generated by interfacial tension. All the samples had the maximum elastic modulus value at 120 min. For the sake of analysis, the elastic modulus value at 120 min was used to present the elastic modulus (ED). In Table 2, the order of ED ranged from high to low: ***β-P*** (61.68 mN/m) > ***121*** (33.65 mN/m) > ***13CG*** (20.37 mN/m) > ***150*** (15.71 mN/m) > ***101*** (14.70 mN/m) > ***104*** (12.70 mN/m). Among the six pectin samples, the ***β-P*** pectin had the highest ED, followed by ***121***. The ***104*** had the lowest ED value (12.70 mN/m). The relatively large ED (61.68 mN/m) of ***β-P*** may be attributed to the formation of a highly viscoelastic interface structure due to forming an interfacial layer by pectin. The increase in ED in the adsorption process was the result of the conformational rearrangement and intermolecular interaction of pectin on the interface, which was similar to the macromolecular substances such as proteins [38]. It was well known that interfacial tension and interfacial viscoelastic can affect the formation and storage stability of emulsions. Some studies have shown that the interfacial tension was negatively correlated with emulsification activity and emulsion stability, while the interfacial viscoelastic was positively correlated [25,39].

### 3.3. Pectin-Based Emulsion

#### 3.3.1. Droplet Characteristics

**Figure 2:**
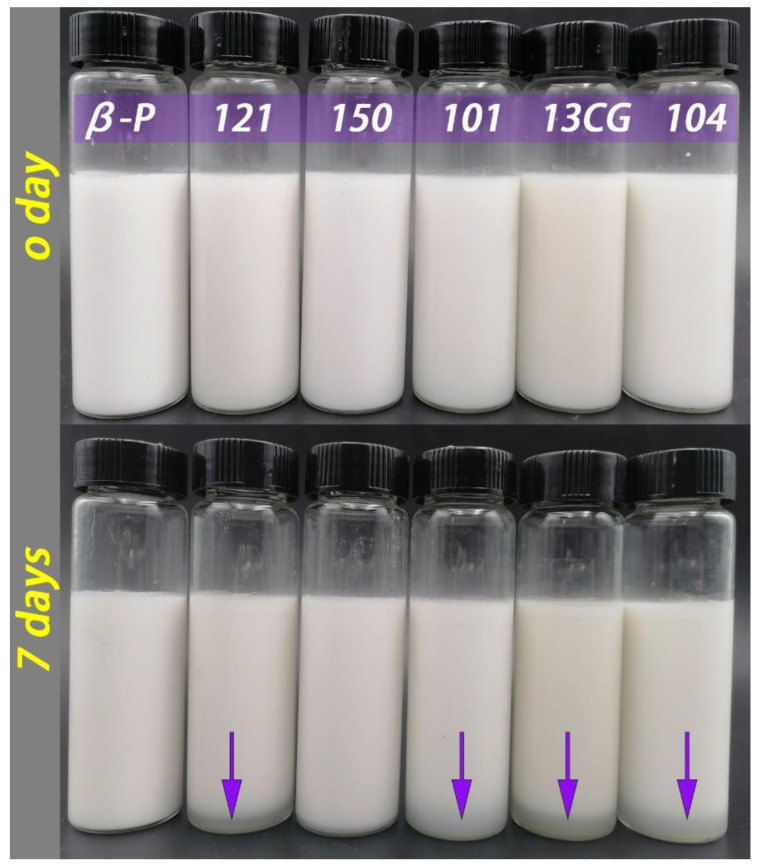
The pectin emulsions (from left to right, ***β-P***, ***121***, ***150***, ***101***, ***13CG,*** and ***104***) were stored at ambient temperature for 0 and 7 days.

**Figure 3:**
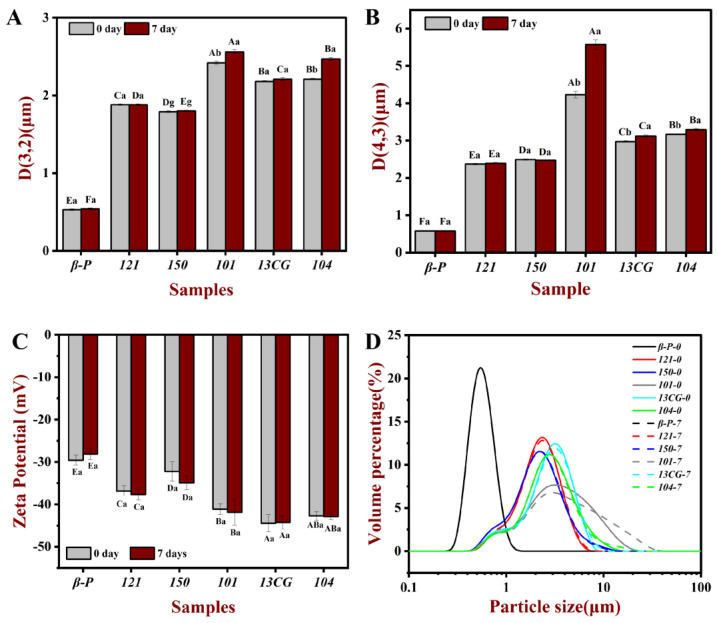
The d_3,2_ (**A**), d_4,3_ (**B**), zeta potential (**C**), and particle size distribution (**D**) of the pectin emulsions (***β-P***, ***121***, ***150***, ***101***, ***13CG****,* and ***104***) stored in ambient temperature for 0 and 7 days, respectively. Note: Different lowercase letters represented that the d_3,2_, d_4,3,_ and zeta potential of the same sample in different storage times had significant differences, different capital letters represented that the d_3,2_, d_4,3_ and zeta potential of different samples in the same storage time had significant difference (*p* < 0.05).

The droplet characteristics include the droplet size, visual evaluation (images), and zeta potential of different pectin emulsions stored for 0 and 7 days. Figure 2 shows the homogeneous milky appearance of all emulsions at 0 days and the appearance of the emulsions after 7-day storage at ambient temperature. After 7 days of storage, ***121***, ***101***, ***13CG,*** and ***104*** showed different degrees of stratification, while ***β-P*** and ***150*** were still stable and without creaming. As shown in Figure 3, the particle size distribution of the pectin emulsion was used to evaluate the forming ability of the emulsion. In Figure 3A, the ***β-P*** emulsion had the smallest particle size value, while ***101*** had the largest particle size value. In Figure 3B, the results of d_4,3_ were consistent with those of d_3,2_, indicating that the best sample was ***β-P*** emulsion and the worst sample was ***104*** emulsion. As shown in Figure 3D, all emulsions have unimodal distribution, indicating that the particle size distribution of the prepared emulsion was relatively uniform, but the particle size distribution of ***β-P*** pectin emulsion was the narrowest. On the contrary, the particle size distribution of ***101*** pectin emulsion was the widest, and the peak was distributed in a larger position, indicating that there were some large oil droplets in the emulsion [5].

Emulsion is a thermodynamically unstable system, a variety of physical mechanisms, including sedimentation, creaming, coalescence, flocculation, and phase inversion, can make the emulsion unstable during the storage time [5]. To evaluate the storage stability of the pectin stabilized emulsion system, the changes of d_3,2_ and d_4,3_ of pectin emulsion during 7 days of storage were determined. As shown in Table 1 and Figure 3, the d_3,2_ and d_4,3_ of ***β-P***, ***121***, and ***150*** pectin emulsions changed little during storage, indicating that the three had relatively good storage stability and ***β-P*** pectin was the best. Within 7 days, the particle size of other pectin stabilized emulsions increased significantly, and the particle size increase in ***104*** pectin emulsion was the highest, indicating that the storage stability of ***104*** pectin emulsion was the worst. This was consistent with the results of the visual evaluation (Figure 2). The ***β-P*** and ***150*** emulsions remained stable for 7 days, but no complete phase delamination was observed in other pectin emulsions; only a very thick cream layer was observed, which can be attributed to bridging flocculation and limited coalescence [40]. It can also be seen from Figure 3D that the particle distribution of ***β-P***, ***121***, and ***150*** pectin emulsions are unchanged, while the particle distribution of other pectin emulsions extends in a larger position after 7 days of storage. The significant difference between the ***101*** and ***β-P*** emulsion may be due to the higher interfacial tension, protein content, and ferulic acid content of ***β-P*** than ***104***. The proteins and the ferulic acid act as a “bridge” between the polysaccharide and oil phases, allowing pectin molecules to act as anchors attached to the water–oil interface, while the carbohydrate portion forms a hydrating layer, which prevents the aggregation or binding of the emulsion droplets [14].

Zeta potential can be used to characterize the stability of the emulsion. The absolute value of zeta potential greater than 30 mV will lead to sufficient repulsive force to stabilize droplets [17]. As shown in Figure 3C, except for ***β-P*** pectin emulsion with a zeta potential slightly greater than -30 mV, all the fresh emulsions prepared from pectin had a low negative charge (less than −30 mV), which indicated that they were electrostatically stable. The DM had a significant effect on the change of zeta potential. The potential of three low-ester pectin was lower than that of high-ester pectin because the lower DM pectin could carry more negative charge [40]. The zeta potential of pectin emulsion was also related to pectin conformation and surface charge density [18], which also indicated that the zeta potential did not change proportionally with the change of DM. It was worth noting that although the zeta potential of ***101***, ***104***, and ***13CG*** emulsions was very small, the particle size of the emulsion was large, which indicated that the zeta potential of the emulsion cannot fully explain its stability. This result can be explained by previous reports that the hydrogen bond between the emulsifier and water may play a crucial role in the stability of the emulsion [41,42] and that changes in negative zeta potential were not directly related to changes in droplet size (emulsion stability) on time scales [43].

#### 3.3.2. Centrifugal Stability

**Figure 4:**
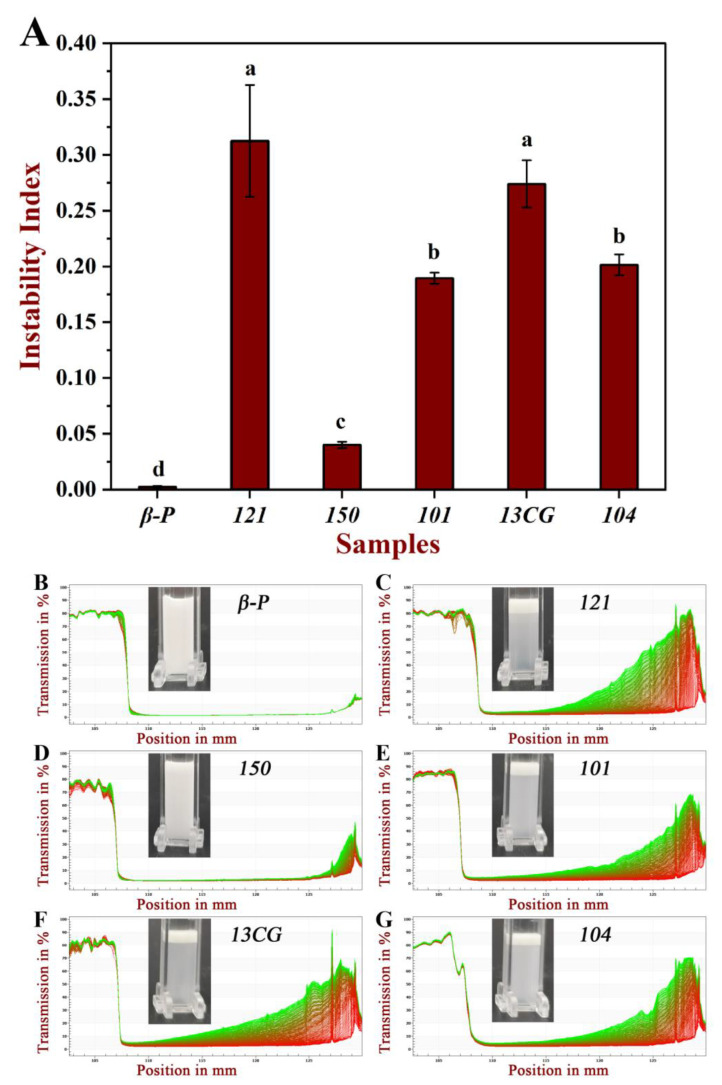
The physical stability of six pectin emulsions. (**A)** The instability index of the pectin emulsions (***β-P***, ***121***, ***150***, ***101***, ***13CG,*** and ***104***). (**B**–**G**) The pectin emulsions (***β-P***, ***121***, ***150***, ***101***, ***13CG,*** and ***104***) on the original transmission profiles over space and time. Note: Different lowercase letters represented that the instability index of different samples had significant difference (*p* < 0.05).

The centrifugal stability of six pectin emulsions was measured by a multi-sample analysis centrifuge based on STEP technology. The physical stability of these emulsions can be reflected by the instability index. Figure 4A shows the instability index of different pectin. The order of the instability index of the six pectin emulsions was: ***121*** (0.313) > ***13CG*** (0.274) > ***104*** (0.202) > ***101*** (0.190) > ***150*** (0.040) > ***β-P*** (0.003). The results showed that the physical stability of ***150*** and ***β-P*** pectin emulsions were significantly higher than the other four pectin emulsions. This may be due to the higher molecular weight and esterification degree leading to the higher viscosity and steric stabilization of ***150*** pectin emulsion, which limited the mobility of dispersed oil droplets and thus inhibited or minimized their migration and binding trend [4,44]. The good stability of ***β-P*** may be due to the extremely high content of ferulic acid, which is hydrophobic and acts as a bridge between oil droplets and polysaccharides [14]. Although ***121*** pectin had the largest protein content, its stability was poor because the emulsifying stability was not simply related to the absolute amount of protein, and the emulsifying performance was affected by the accessibility of protein to the surface of the oil droplets [12]. Figure 4B–G further showed the six pectin emulsions on the original transmission profiles over space and time. The position in the transmission profiles at about 107 mm corresponded to the filling height of the emulsions, and the position of the cell bottom was at 130 mm. The first profile lay was the red section at the bottom, and the last profiles lay was the green section at the top. Figure 4B further proved that the transmittance of ***β-P*** and ***150*** pectin emulsions varies much less with time and space than the other four emulsions, especially for ***β-P*** emulsions, whose profiles are almost completely coincident, indicating good physical stability.

#### 3.3.3. The Rheological Properties of Emulsion

**Figure 5:**
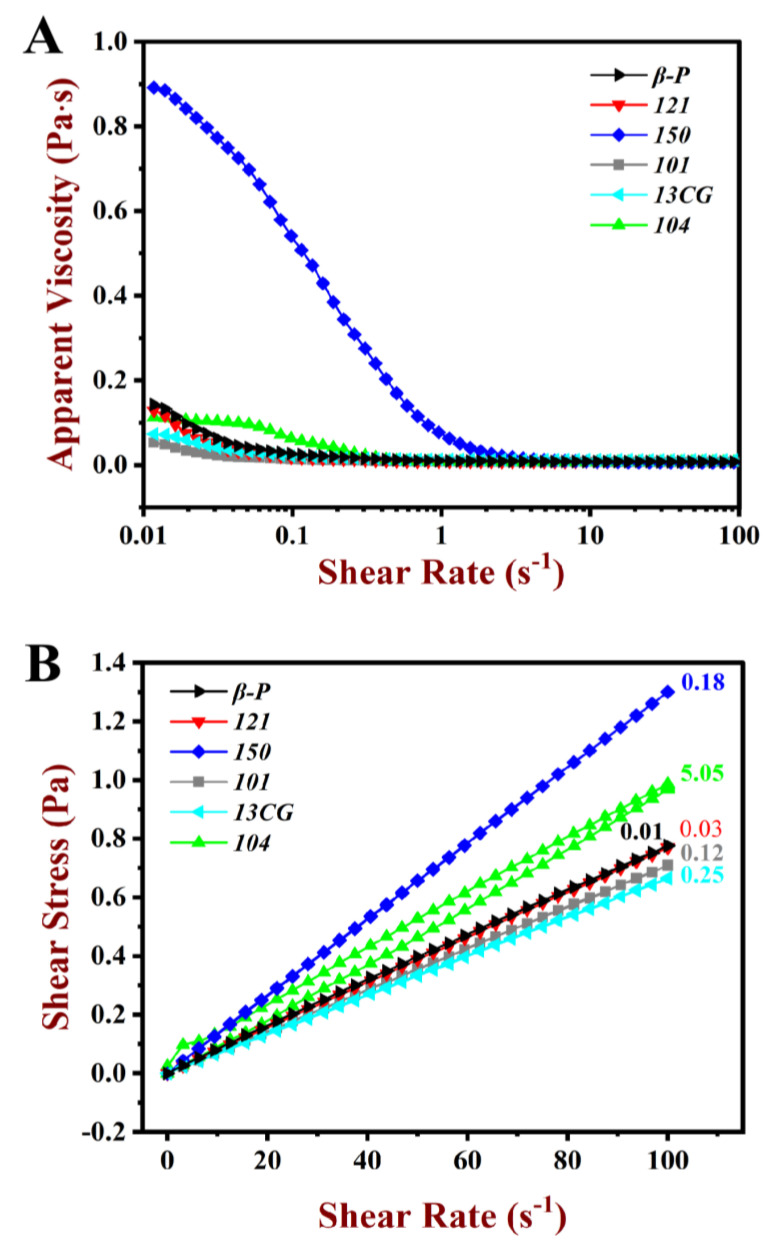
Steady rheological behavior of the pectin emulsions. (**A**) Apparent viscosity of the ***β-P***, ***121***, ***150***, ***101***, ***13CG,*** and ***104*** emulsions. (**B**) Shear stress of the ***β-P***, ***121***, ***150***, ***101***, ***13CG,*** and ***104*** emulsions.

The rheological properties of pectin emulsions, including apparent viscosity and shear stress, are shown in Figure 5. As shown in Figure 5A, for the pectin emulsions, apparent viscosity decreased as the shear rate increased, suggesting the shear-thinning behavior of the pseudo-plastic fluid. At low shear viscosity, relatively low apparent viscosity was observed in most pectin emulsions except for ***150***. The emulsion stabilized by ***150*** had relatively high apparent viscosity and DM, which may attribute to the large Mw of ***150*** pectin [45]. The M_W_ of ***150*** pectin was 1273 kDa, which was remarkably higher than other pectins (lower than 887 kDa). Related studies showed that the effect of viscosity on emulsification was twofold. On the one hand, higher viscosity may limit the absorption rate of pectin molecules during emulsification. On the other hand, high emulsion viscosity helps to inhibit the accumulation of emulsions and droplets [46]. Evidently, the latter had a greater influence on ***150*** emulsion, and the high viscosity ensured the stability of the emulsion.

In Figure 5B, all the pectin emulsions were sheared from 0.01 to 100 s^−1^, and subsequently, they were sheared again from 100 to 0.01 s^−1^. Stress-shear hysteresis loops area (HLA) were observed during this shear process. A large HLA indicated a strong thixotropic effect. The ***104*** emulsion had larger HLA than the other pectin emulsions, which indicated that the emulsions had large shear thixotropy [47]. As shown in Table 2, the ***121*** and ***β-P*** emulsions had little HLA (<0.04 Pa·s), which indicated that the two pectin emulsion structures were unsusceptible to the destroy in the shear process.

#### 3.3.4. The Digestive Behavior of Emulsion

**Figure 6:**
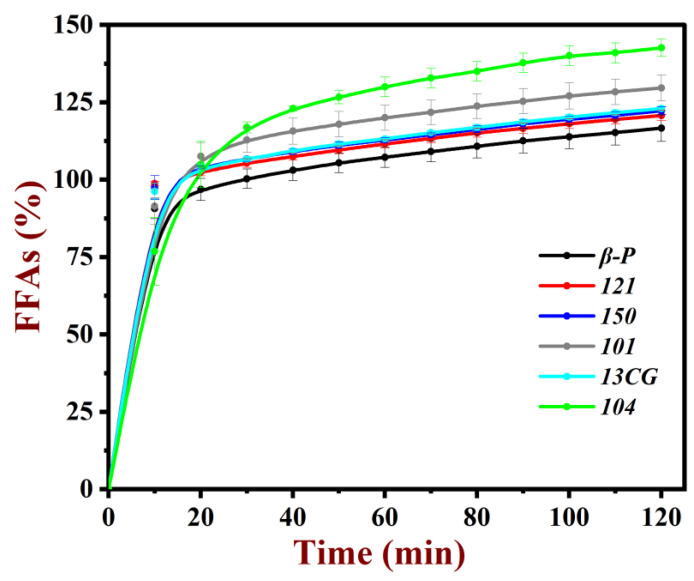
Amount of free fatty acid (FFA) released from pectin emulsions.

The oil in emulsions will be broken down by the lipase at the small intestine and changed to free fatty acids. The amount of free fatty acids (FFAs) released was calculated to provide information about the digestive behavior of pectin emulsion. As shown in Figure 6, all the pectin emulsions had relatively fast fat hydrolysis rates at the first 10 min. Then, the hydrolysis rates reached a balance as the digestive time. After 2 h, all the oil in the emulsion had been broken down adequately. As shown in Table 2, the amount of free fatty acids (FFAs) released was compared: ***104*** (142.6%) > ***101*** (129.6%) > ***13CG*** (123.0%) ≈ ***150*** (122.2%) ≈ ***121*** (120.7%) ≈ ***β-P*** (116.6%). It was worth noting that the FFAs of low methoxyl pectin (***104***, ***101***, ***13CG***) emulsions were larger than FFAs of high methoxyl pectin (***150***, ***121***, and ***β-P***) emulsions. This result indicated the DM of pectin had a significant effect on the pectin emulsions [48]. It was well-known that the esterification of pectin can improve the hydrophobicity and reduced the negative charge of pectin. Pectin with more hydrophobicity was more susceptible to binding to bile salts, then hindered the absorption of fat for enzymes and ultimately inhibited the digestion of fat [49].

## 4. Conclusions

Pectin is a new type of natural polymer emulsifier which has been widely used in the food industry. In order to fully understand the emulsifying properties of pectin, it was necessary to comprehensively analyze the effects of pectin structure on emulsion emulsification, rheological properties, and in vitro digestibility. The results showed that the ***β-P*** pectin had the highest acetyl and ferulic acid content, and the emulsion formed by ***β-P*** pectin exhibited smaller interfacial tension and particle size and better physical stability. However, the ***150*** pectin had the highest molecular weight and DM, the emulsion of which had higher viscosity, restraining the aggregation of emulsion droplets to some extent. In addition, the degree of methoxylation of pectin can significantly affect the digestion behavior, showing that the amount of free fatty acid released from low-ester pectin emulsions was higher than that of high-ester pectin. These results provide new insight into the rational selection and structural design of pectin emulsifiers.

## Figures and Tables

**Table 1:** The structural properties of different pectin.

Sample	GA (%)	M_W_ (kDa)	DM (%)	DAm (%)	DAc (%)	Protein (%)	Ferulic Acid (%)
***β-P***	81.02 ± 1.29 ^c^	714 ± 11 ^d^	62.46 ± 0.72 ^b^	N/A	3.03 ± 0.02 ^a^	4.67 ± 0.01 ^b^	1.43 ± 0.01 ^a^
***121***	82.29 ± 2.34 ^b^	623 ± 21 ^e^	58.48 ± 2.31 ^c^	N/A	2.73 ± 0.05 ^b^	7.74 ± 0.11 ^a^	0.25 ± 0.01 ^c^
***150***	78.33 ± 0.91 ^d^	1273 ± 142 ^a^	72.56 ± 3.62 ^a^	N/A	2.61 ± 0.08 ^b^	4.42 ± 0.09 ^c^	0.34 ± 0.01 ^b^
***101***	70.17 ± 0.57 ^e^	887 ± 13 ^b^	37.13 ± 0.77 ^e^	14.89 ± 0.29 ^b^	2.33 ± 0.17 ^c^	2.65 ± 0.18 ^e^	0.24 ± 0.01 ^c^
***13CG***	87.73 ± 1.33 ^a^	527 ± 67 ^f^	41.46 ± 0.42 ^d^	N/A	2.31 ± 0.08 ^c^	3.47 ± 0.03 ^d^	0.25 ± 0.01 ^c^
***104***	65.59 ± 0.78 ^f^	786 ± 78 ^d^	28.28 ± 1.31 ^f^	20.63 ± 0.51 ^a^	2.40 ± 0.02 ^c^	1.90 ± 0.39 ^f^	0.18 ± 0.01 ^d^

Note: The mean values in the same column with different letters have significant difference (*p* < 0.05); N/A indicates that the content did not reach the detection limit.

**Table 2:** The physicochemical properties of different pectin or pectin-based emulsions *.

Sample	γ (mN/m)	ED (mN/m)	d_3,2_ (μm)	∆d_3,2_ (μm)	d_4,3_ (μm)	∆d_4,3_ (μm)	HLA (Pa/s)	FFAs (%)
***β-P***	14.89	61.68	0.53 ± 0.01 ^e^	0.54 ± 0.01 ^f^	0.58 ± 0.01 ^f^	0.58 ± 0.01 ^f^	0.01	116.65 ± 4.34 ^d^
***121***	17.27	33.65	1.88 ± 0.01 ^c^	1.88 ± 0.01 ^d^	2.37 ± 0.02 ^e^	2.39 ± 0.02 ^e^	0.03	120.72 ± 1.68 ^cd^
***150***	18.62	15.71	1.79 ± 0.01 ^d^	1.8 ± 0.01 ^e^	2.49 ± 0.02 ^d^	2.47 ± 0.01 ^d^	0.18	122.22 ± 1.30 ^cd^
***101***	18.69	14.70	2.42 ± 0.02 ^a^	2.56 ± 0.03 ^a^	4.23 ± 0.09 ^a^	5.57 ± 0.13 ^a^	0.12	129.63 ± 4.19 ^b^
***13CG***	19.02	20.37	2.18 ± 0.01 ^b^	2.21 ± 0.02 ^c^	2.97 ± 0.02 ^c^	3.12 ± 0.03 ^c^	0.25	122.95 ± 0.80 ^c^
***104***	21.02	12.70	2.21 ± 0.01 ^b^	2.47 ± 0.02 ^b^	3.17 ± 0.01 ^b^	3.29 ± 0.03 ^b^	5.05	142.58 ± 2.76 ^a^

* The mean values in the same column with different letters have significant difference (*p* < 0.05), the γ present the interfacial tension of pectin at 180 min, the ED present the elastic modulus at 120 min, d_3,2_ and d_4,3_ represent the surface-weighted average diameter and volume weighted average diameter of fresh emulsion, respectively, and ∆d_3,2_ and ∆d_4,3_ represent the change of surface-weighted average diameter and volume weighted average diameter of fresh emulsion during the storage time of 7 days, respectively, and the FFAs present the free fatty acids of emulsion at 120 min.

## Data Availability

Data are contained within the article.
